# Supplementary material for: Identification of Src as a Therapeutic Target in Oesophageal Adenocarcinoma through Functional Genomic and High-Throughput Drug Screening Approaches
Source: Cancers (Basel). 2022 Jul 30;14(15):3726. doi: 10.3390/cancers14153726 (PMC9367554; doi:10.3390/cancers14153726)
Supplement: Supplementary file 1 [file cancers-14-03726-s001.zip › cancers-1737089-supplementary.pdf]

Supplementary Table S2. OAC cell line drug sensitivity as assessed by MTT assay following a 72 h dose response from which ~$IC_{30(72h)}$ doses (μM) were calculated.

**OAC Cell Line**	**CDDP**	**5-FU**	**Dasatinib**	**Saracatinib**
OE33	1	0.5	0.025	0.5
FLO-1	3.5	5	0.005	3
SKGT4	1.5	4	0.075	0.075
OE19	10	14	0.25	0.25
MFD-1	1	5	0.03	0.5
OE33-CDDP-R	4			

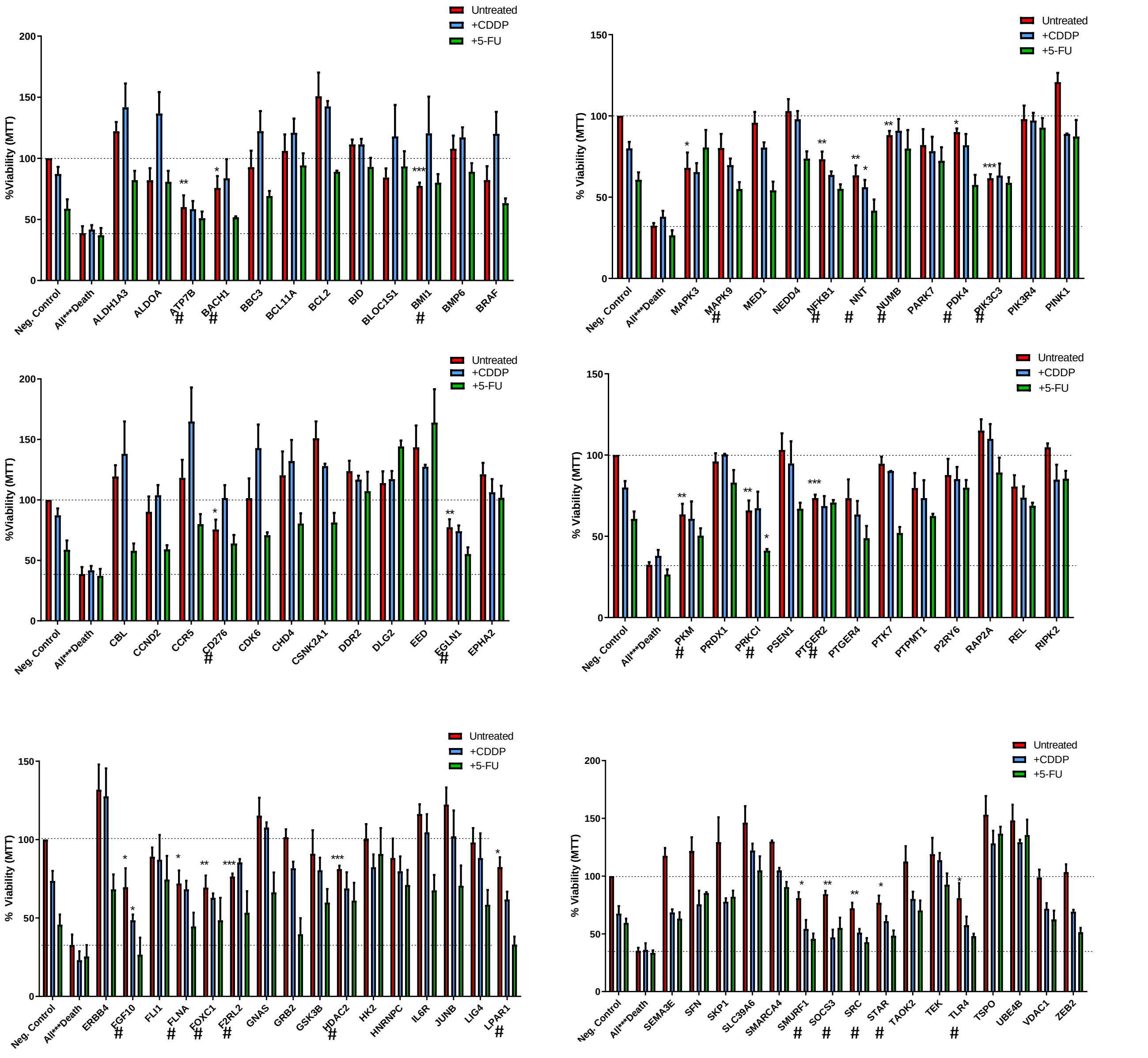

Supplementary Figure S1a. Primary RNAi screen. A set of 80 selected target genes were investigated for their functional effect in OAC. For this, OE33 cells were transfected with 10 nM siRNA for 24 h followed by ± 48 h treatment with ~$IC_{30(72h)}$ CDDP or 5-FU. Viability was assessed using MTT assays and Student's t-test was applied to test for significance with *p<0.05, **p<0.01, ***p<0.001 and ns (non-significant). Results represent the mean of triplicate experiments ± SEM. # Positive hits carried forward are highlighted.

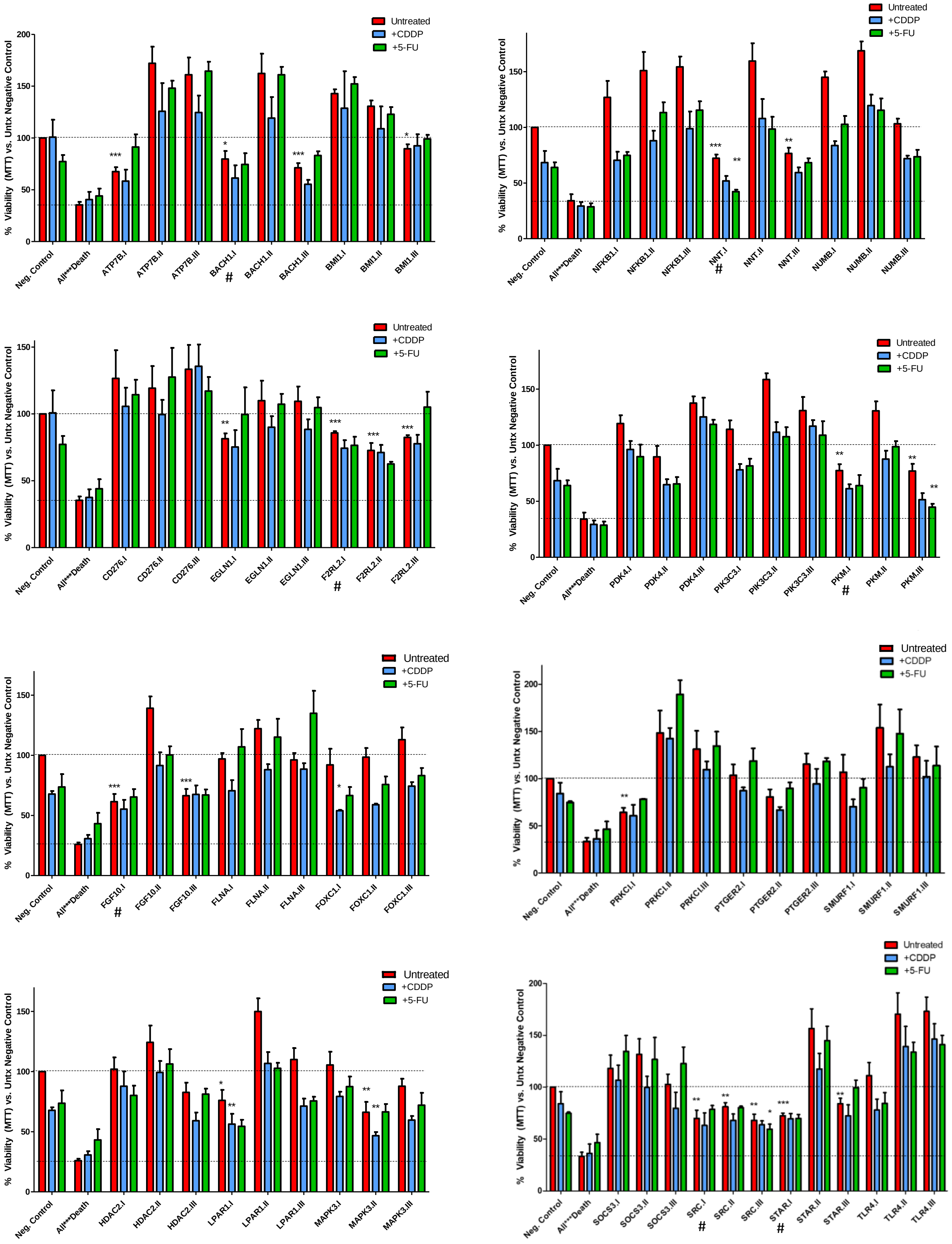

Supplementary Figure S1b. Secondary RNAi screen. A set of 25 genes identified as positive hits from the primary RNAi screen were further investigated. For this, OE33 cells were transfected with 3 siRNAs per target gene at 10 nM siRNA for 24 h followed by ± 48 h treatment with ~$IC_{30(72h)}$ CDDP or 5-FU. Viability was assessed using MTT assays and Student's t-test was applied to test for significance with *p<0.05, **p<0.01, ***p<0.001 and ns (non-significant). Results represent the mean of triplicate experiments ± SEM. # Positive hits carried forward are highlighted.

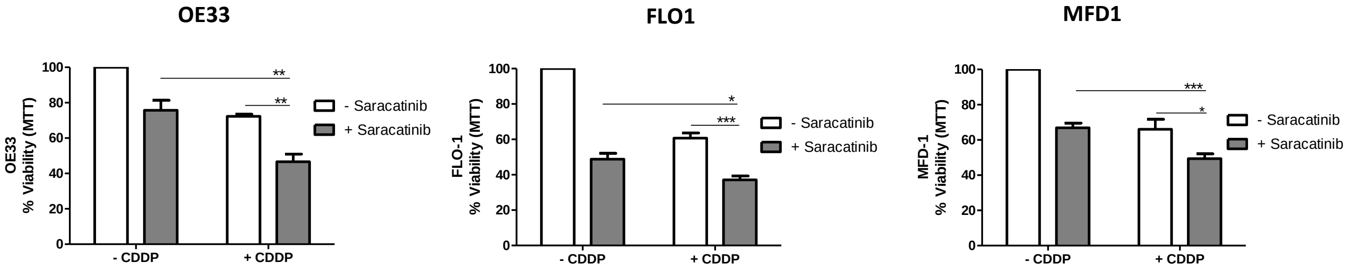

(a)

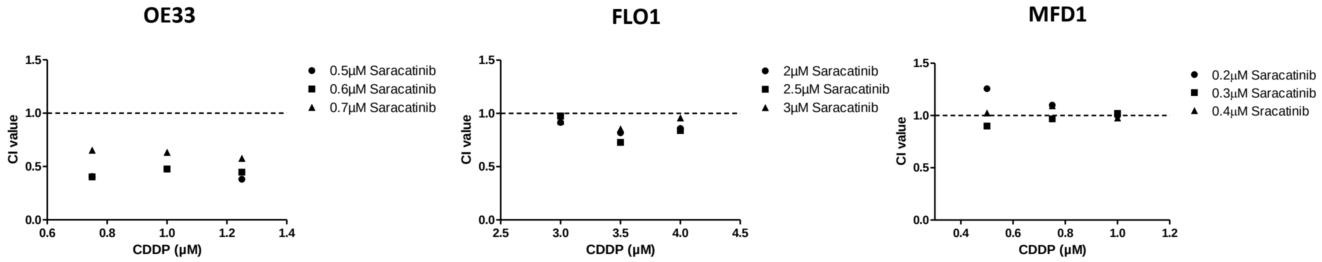

(b)

Supplementary Figure S2. Saracatinib synergises with CDDP in OAC cell lines. (a) OE33, FLO-1 and MFD-1 cells were treated with saracatinib/CDDP combinations for 72 h at ~$IC_{30(72h)}$ doses. (b) Saracatinib/ CDDP drug interactions were assessed by generating CI values using the Chou & Talalay method. Viability was assessed throughout using MTT assays. Student's t-test was applied to test for significance with *$p<0.05$, **$p<0.01$, ***$p<0.001$ and ns (non-significant). Results represent the mean of triplicate experiments ± SEM. CI<1 indicates synergy, CI=1 indicates additive and CI>1 indicates an antagonistic interaction.

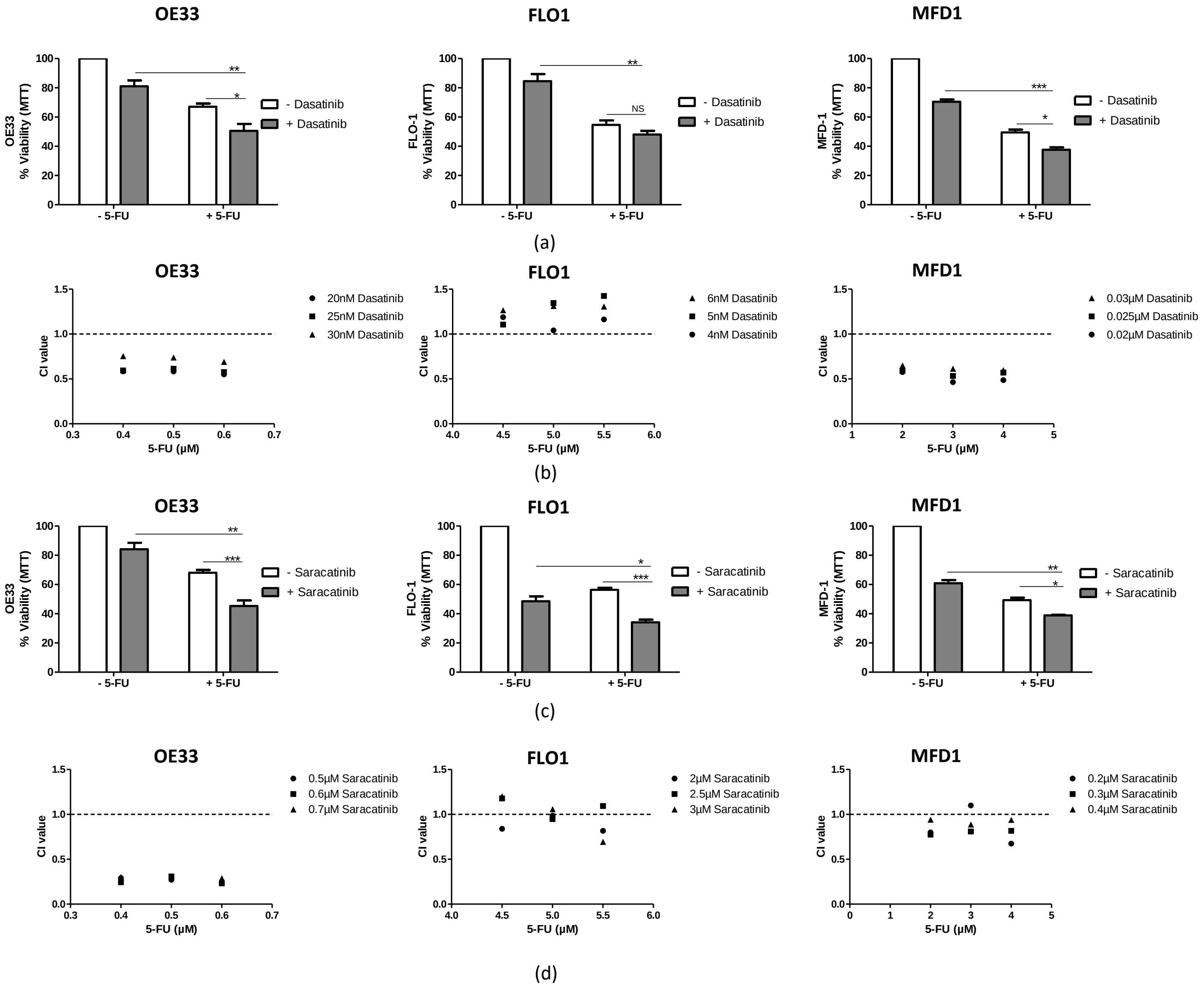

Supplementary Figure S3. Src inhibition synergises with 5-FU in OAC cell lines: (a) OAC cell lines OE33, FLO-1 and MFD-1 were treated with dasatinib/5-FU for 72 h at ~$IC_{30(72h)}$ doses. Viability was assessed using MTT assays and Student's t-test was applied to test for significance with *p<0.05, **p<0.01, ***p<0.001, and ns (non-significant). Results represent the mean of triplicate experiments ± SEM. (b) Viability was assessed using MTT assays following 72 h combination treatments with multiple drug doses ≤~$IC_{30(72h)}$. Dasatinib/5-FU drug interactions were assessed by generating combination index (CI) values using the Chou & Talalay method where a CI<1 indicates synergy, CI=1 indicates additive and CI>1 indicates an antagonistic interaction. (c) OAC cell lines OE33, FLO-1 and MFD-1 were treated with saracatinib/5-FU for 72 h at ~$IC_{30(72h)}$ doses. Viability was assessed using MTT assays and Student's t-test was applied to test for significance with *p<0.05, **p<0.01, ***p<0.001, and ns (non-significant). Results represent the mean of triplicate experiments ± SEM. (d) Viability was assessed using MTT assays following 72 h combination treatments with multiple drug doses ≤~$IC_{30(72h)}$. Saracatinib/5-FU drug interactions were assessed by generating combination index (CI) values using the Chou & Talalay method where a CI<1 indicates synergy, CI=1 indicates additive and CI>1 indicates an antagonistic interaction.

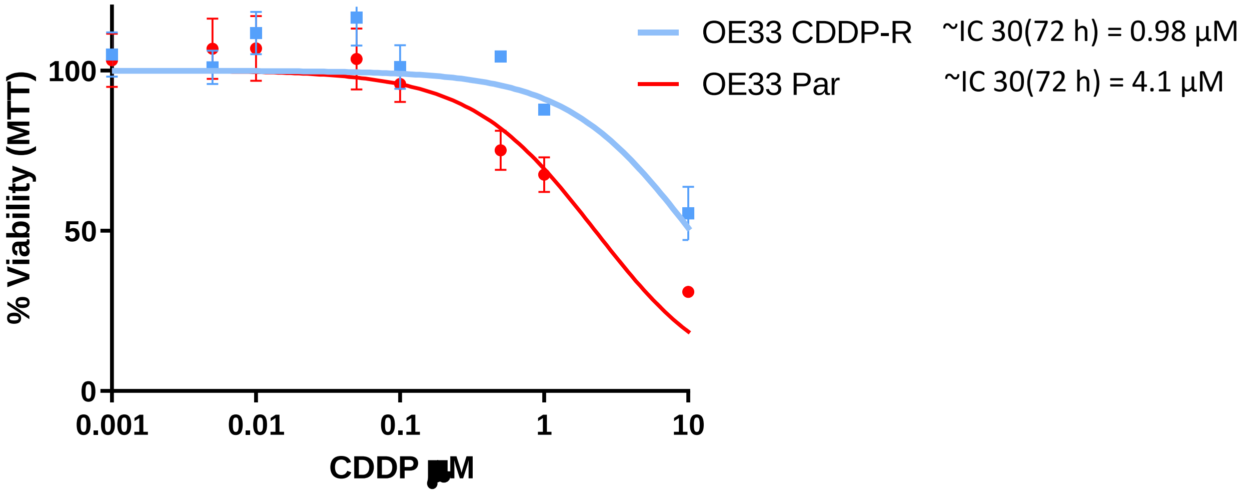

Supplementary Figure S4. CDDP dose response in OE33 parental and CDDP-R cell line pair. MTT assay was applied to measure viability at 72 h post CDDP treatment and subsequently generate a dose response curve using PRISM software. Triplicate results are represented as Mean ± SEM.

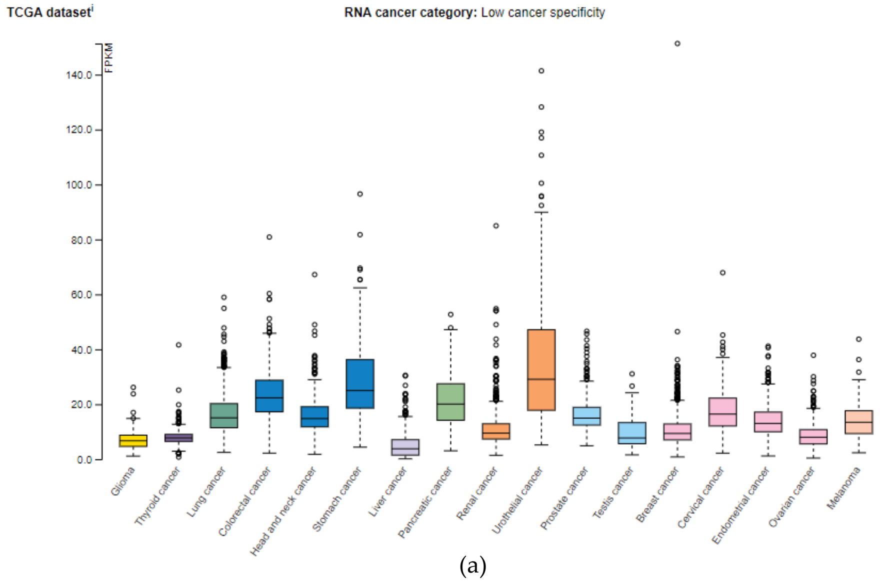

(a)

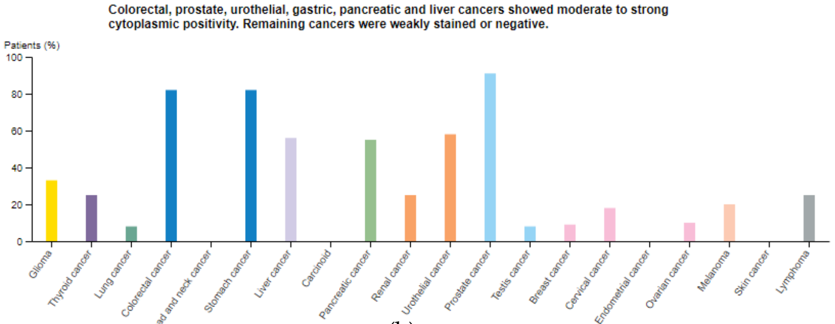

(b)

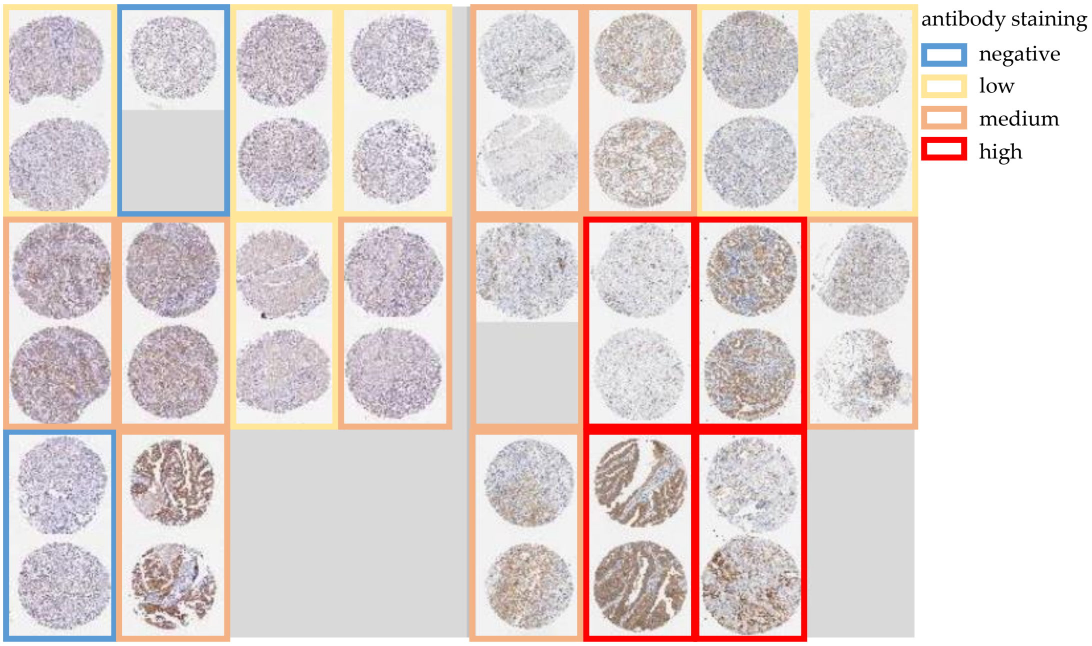

(c)

Supplementary Figure S5. Src expression data of gastric adenocarcinoma (https://www.proteinatlas.org/ENSG00000197122-SRC/pathology). (a) The Cancer Genome Atlas (TCGA) RNA-seq data in 17 cancer types are reported as median FPKM (number Fragments Per Kilobase of exon per Million reads), generated by the The Cancer Genome Atlas (TCGA). RNA cancer tissue category is calculated based on mRNA expression levels across all 17 cancer tissues and include: cancer tissue enriched, cancer group enriched, cancer tissue enhanced, expressed in all, mixed and not detected. Normal distribution across the dataset is visualized with box plots, shown as median and 25th and 75th percentiles. Points are displayed as outliers if they are above or below 1.5 times the interquartile range. The cancer types are color-coded according to which type of normal organ the cancer originates from. (b) Percentage of tumours showing high/medium expression of Src protein across 16 cancer types. Sections were stained with Src antibody CAB004023. (c) Tissue sections of Src protein expression in gastric adenocarcinoma tissue sections from 21 stomach cancer patients. Stomach cancer protein expression. Left hand panel of sections were stained with antibody HPA030875. Right hand panel of sections were stained with antibody CAB004023.
